# Study on the Impact Failure Rules of Roadway Floor Induced by Dynamic Load Disturbance under Extremely Thick Conglomerate

**DOI:** 10.1021/acsomega.4c00736

**Published:** 2024-05-24

**Authors:** Xuefeng Xu, Zuo Dai, Hao Xu

**Affiliations:** † School of Energy Science and Engineering, Henan Polytechnic University, Jiaozuo 454000, People’s Republic of China; ‡ Key Laboratory of Green and Efficient Mining and Comprehensive Utilization of Mineral Resources in Henan Province, Henan Polytechnic University, Jiaozuo 454000, People’s Republic of China; § College of Safety and Environmental Engineering, Shandong University of Science and Technology, Qingdao 266590, People’s Republic of China

## Abstract

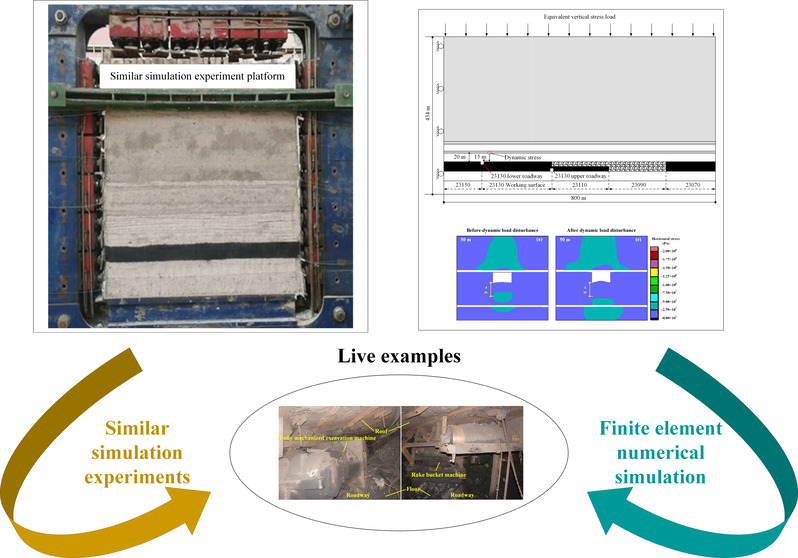

The dynamic load resulting from the fracture of extremely thick rock layers directly influences the surrounding rock layers within stopes and roadways, thereby inducing rockburst disasters. Hence, studying the tunnel floor’s impact and ground pressure induced by dynamic load disturbance under extremely thick conglomerates is crucial. This study focuses on the 23130 working face of Yuejin Coal Mine as its engineering background. Initially, through similar simulation experiments, the impact characteristics of dynamic load disturbance positions under thick conglomerates on tunnel bottom damage are investigated. Building upon this foundation, finite element numerical simulation is employed to explore the further conglomerate thickness’ impact on tunnel floor damage under dynamic load disturbance. Lastly, the accuracy of similar and numerical simulation results is validated by incorporating field examples. Findings reveal that dynamic load disturbance leads to an instantaneous increase in coal and rock mass acceleration in the roof and floor of roadways, followed by a decrease to an equilibrium state, thereby subjecting the interior to high static load conditions. The thickness of conglomerate in the overlying rock layer emerges as a crucial factor affecting tunnel floor rockburst incidents. With dynamic load disturbance, as conglomerate thickness increases, the stress concentration area of the tunnel floor gradually shifts to deeper rock strata. Effective control of tunnel floor rockbursts can be achieved by implementing support measures like anchor rods and cables and managing tunnel deformation and damage under dynamic loads. Dynamic load disturbance under extremely thick conglomerates emerges as a pivotal condition for inducing tunnel floor impact damage. This study provides a theoretical foundation for the safe excavation of similar mine tunnels and for implementing rockburst prevention and control measures.

## 1 Introduction

Coal is an indispensable conventional energy in many countries and regions. Ensuring safe mining and efficient operation of coal mines has always been the long-term goal of the coal industry.^1,2^ The complex geological environment and tectonic movement of the coal seam determine that the underground coal seam mining process will face potential risks such as water inrush, rockburst, gas outburst, and coal dust explosion.^3,4^ In China, coal consumption still constitutes 65% of total primary energy consumption.^5^ In recent years, there has been a notable escalation in the depth and intensity of coal mining operations, further elevating the likelihood of mine disasters such as rockbursts.^6^

Rockburst, the most severe dynamic damage disaster in deep mines, primarily occurs due to the release of elastic strain energy from the rock mass surrounding mine tunnels or stopes. When stress becomes concentrated within the rock mass, the stored energy is suddenly released, resulting in powerful dynamic loads that cause significant damage to the surrounding rock, leading to casualties and equipment damage.^7,8^ Currently, numerous experts and scholars have researched rockbursts from various perspectives. Wang et al.^9^ proposed a practical rockburst analysis method combining dynamic and static loads and assessed the rockburst risk of longwall mining faces. Cao et al.^10^ selected rockburst cases and studied the entire failure process of rockbursts through large-scale numerical simulations, revealing the disaster-incubating mechanism of rockbursts. Kong et al.^11^ systematically investigated the evolution mechanism and hazard of rockburst events induced by fault slip under the influence of dynamic loads using various research methods, including field investigations, physical experiments, theoretical analysis, and numerical simulations. Song et al.^12^ established a dynamic ejecting coal blasting model of coal mine roadway under stress and believed that the stress concentration area on the roadway side was the direct energy source of the ejecting. Through the coupling analysis of theoretical analysis, numerical simulation, and physical experiment, Cai et al.^13^ determined that the interaction between discontinuous structure and mining activity is the key factor that dominates the reactivation of faults and may induce rockburst.

The causes of rockburst are highly complex, involving not only the physical and mechanical properties and stress conditions of coal and rock masses but also various mining activities such as coal blasting and roof collapse that disrupt the equilibrium state of the tunnel.^7,14−16^ Throughout the mining process, dynamic load disturbances inevitably occur, leading to the simultaneous presence of dynamic and static loads, which fundamentally contribute to rockbursts.^17,18^ The significant thickness, high hardness, and extensive roof span are primary factors contributing to rockbursts and similar disasters.^19^ Thick and rugged rock formations typically encompass sandstone, conglomerate, igneous rock, and other rock masses characterized by strong integrity, high strength, and robust self-stability.^20,21^ When a thick and hard rock layer lies atop the working surface, it not only accumulates elastic energy efficiently but also serves as a solid medium for effective high-stress transmission. Thus, fractures within such rock layers directly influence the occurrence of rockbursts induced by internal rock layers surrounding the slope and roadway.^22,23^ Numerous experts and scholars have explored the relationship between overlying complex rock formations and rockbursts from various perspectives. He et al.^24^ studied the mechanism and type of rockburst induced by hard roof by means of mechanical model and numerical simulation of surrounding rock. By studying the failure modes of different hard coal-rock combinations, Yang et al.^25^ and Chai et al.^26^ obtained that the pulse stress formed by the fracture of the hard roof can easily cause the sudden instability of the energy storage coal body, resulting in the occurrence of rockburst. Du et al.^27^ used multiple coupling methods of theoretical analysis, field monitoring, numerical simulation, and engineering verification to explore the mechanism of rockburst in a fully mechanized caving face under the condition of coal seam left behind by a hard roof. He et al.^28^ simulated the complete process of rockburst induced by mine tremors using UDEC discrete element numerical simulation, revealing the entire dynamic process from generation and propagation to triggering of rockbursts caused by mine tremors and exploring the rockburst risks under different geological conditions.

In summary, while there is an established correlation between rockburst occurrences and the thickness of overlying hard rock, more research still needs to be done on tunnel floor impact ground pressure induced by dynamic load disturbance under extremely thick conglomerates. Thus, this study focuses on the 23130 working faces of the Yuejin Coal Mine in the Yima Coalfield, Henan Province. By establishing a similar simulation platform for assessing tunnel floor damage caused by dynamic load disturbance under significant gravel thickness, we explore the impact characteristics of dynamic load disturbance position on tunnel floor damage. Building upon this foundation, a numerical simulation model was developed using FLAC 2D finite element numerical simulation software. By analyzing stress changes and tunnel floor damage degree, we investigate how different conglomerate thicknesses affect tunnel floor damage under a dynamic load disturbance. Finally, through field examples, we demonstrate the significant contribution of dynamic load disturbance under extremely thick conglomerates to tunnel floor impact damage. This research provides a theoretical framework for safe excavation practices in similar mine tunnels. It informs the implementation of effective rockburst prevention and control measures, thereby enhancing the safety and efficiency of mining operations.

## 2 Engineering Background Overview

**Figure 1:**
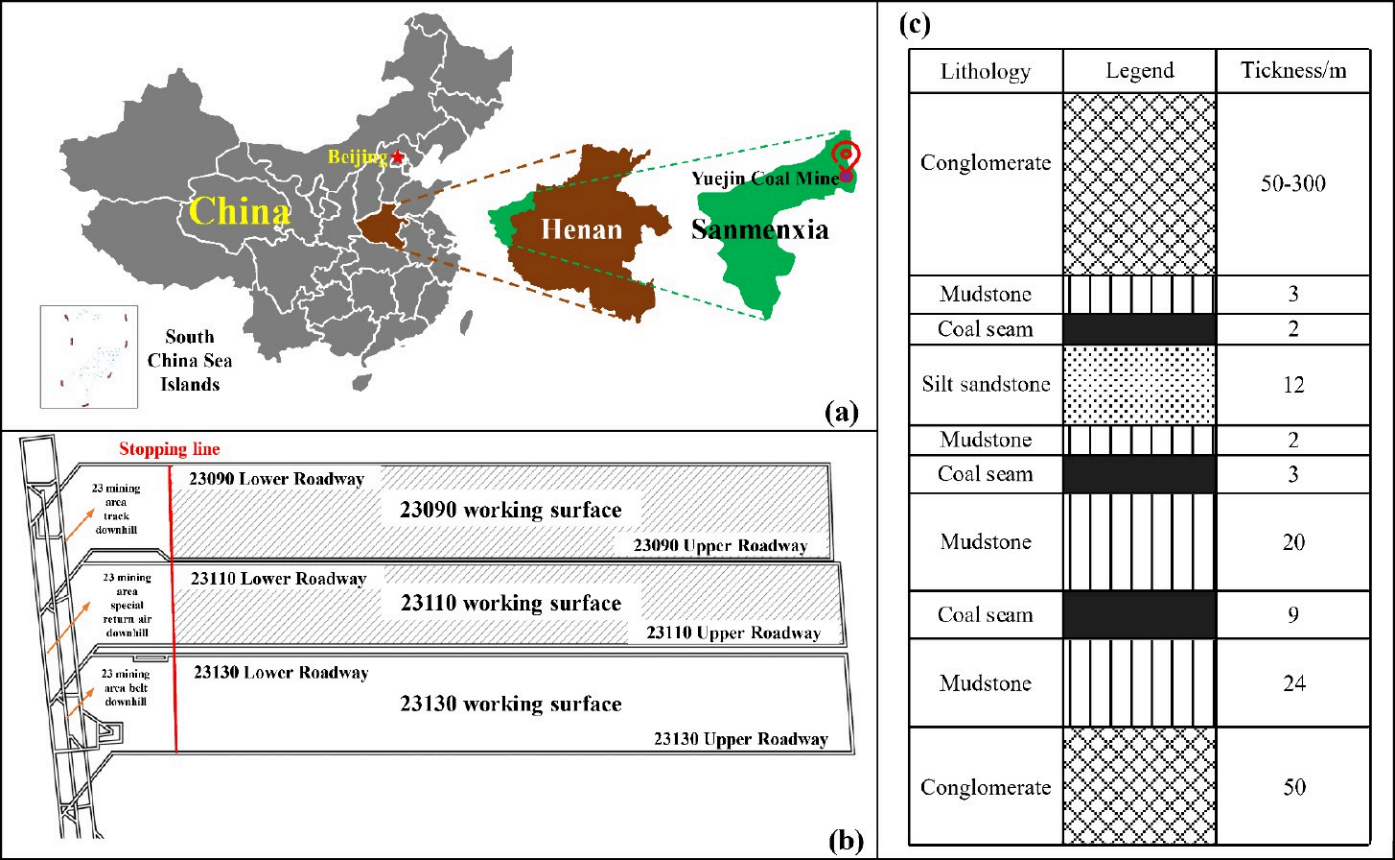
Engineering Project overview basic drawing. (a) Location map of Yuejin Coal Mine. (b) 23130 working surface layout drawing. (c) Geological column diagram of 23 mining areas. Adapted or reprinted in part with permission from [WangG.; FanC.; XuH.; LiuX.; WangR.Determination of long horizontal borehole height in roofs and its application to gas drainage.Energies2018, 11 ( (10), ), 2647]. Copyright [2018] [MDPI/Wang, G].

The Yima Coalfield, situated in Mianchi County, Yima City, Henan Province, comprises five operational coal mines. These include Yangcun, Gengcun, Qianqiu, Yuejin, and Changcun Coal Mines, arranged from west to east. The terrain of Yuejin Coal Mine is characterized by hills and mountains, with the 23130 working face situated in the 23 mining area at a depth of 800–1000 m. Figure 1 illustrates the layout and geological conditions of the working face and its adjacent areas. One notable feature of the overlying rock formation on the working surface is an ultrathick conglomerate layer, which can reach depths of ten to hundreds of meters. Composed primarily of quartzite and quartz sandstone, with gravel particle sizes ranging from 2 to 500 mm, this layer has the potential to store significant elastic energy.^29^ As mining operations progress and the goaf area expands, the accumulation of elastic energy within the ultrathick gravel layer poses an increasing risk of rockbursts within the tunnel. This presents significant challenges in controlling and mitigating rockburst incidents within the tunnel.

The 23130 working face employs fully mechanized caving mining technology, with the working face tunnel adopting an internal staggered arrangement. The upper tunnel of the 23130 working face is positioned above the second layer of the 23110 working face. In comparison, the lower tunnel of the 23130 working face is excavated along the roof of the coal seam, leaving the bottom coal intact. The dimensions of the tunnel measure 4 × 3 m, with both sides and the roof supported by anchor rods. However, the bottom plate does not utilize any support method. Throughout the excavation process of the 23130 working faces, 7 floor impact incidents occurred, with 1 incident occurring during the mining process.

## 3 A Similar Simulation Experiment on the Impact of Dynamic Load Disturbance under Extremely Thick Conglomerate

### 3.1 Similar Model Making

#### 3.1.1 Material Ratio

The similarity simulation experiment on mine pressure should satisfy geometric, motion, dynamic, stress, external force, stress–strain relationship, strength curve, and time characteristic similarities.^30^ To ensure the stability of the model, the test model frame for similarity simulation needs to have adequate stiffness and a certain width. Therefore, based on the available test conditions, a rigid model frame measuring 1600 mm × 400 mm × 1600 mm (length × width × height) was selected for the test.

According to the similarity principle of Newtonian mechanics,^31^ and considering the size of the selected model frame and other comprehensive conditions, the geometric similarity constant of the model is determined to be *C*_l_ = 100. Given that the bulk density of the rock mass is 2.6 g/cm^3^, and the density of the similar material consolidation is 1.5 g/cm^3^, the density similarity constant of the model material is calculated as *C*_p_ = 2.6/1.5 = 1.73. Consequently, the stress similarity constant is obtained as *C*_σ_ = *C*_l_ × *C*_p_ = 100 × 1.73 = 173. A comparison of the compressive strength and bulk density of the original rock and the model is presented in Table 1.

**Table 1:** Mechanical Parameters of Original Rock and Model

no.	lithology	thickness (mm)	prototype compressive strength (MPa)	prototype bulk density (g/cm^3^)	model compressive strength (MPa)	model bulk density (g/cm^3^)
1	conglomerate	800	52.05	2.6	0.301	1.50
2	mudstone	30	20	2.5	0.116	1.45
3	coal seam	20	14.43	1.35	0.083	0.78
4	silt sandstone	120	35.25	2.6	0.204	1.50
5	coal seam	30	14.43	1.35	0.083	0.78
		20	20	2.6	0.116	1.45
		20	20	2.6	0.116	1.45
		20	20	2.6	0.116	1.45
		20	20	2.6	0.116	1.45
6	mudstone	20	20	2.6	0.116	1.45
		20	20	2.6	0.116	1.45
		20	20	2.6	0.116	1.45
		20	20	2.6	0.116	1.45
		20	20	2.6	0.116	1.45
		20	20	2.6	0.116	1.45
7	coal seam	100	14.43	1.35	0.083	0.78
8	mudstone	240	20	2.6	0.116	1.45

In this similarity test, sand was utilized as aggregate, while calcium carbonate and gypsum served as cementing materials, with borax employed as a retarder. The similar simulation experiment of mine pressure and relevant literature determined a reasonable ratio of similar materials in each layer.^32^ After laying each layer, mica powder was sprinkled to ensure clear bedding between the model coal seam and the rock layer. Considering the cross-sectional area of the model frame, the coal seam and rock layer thickness, and the geometric similarity ratio, the volume of each similar material required could be calculated. As illustrated in Table 2, with an abundance coefficient of 1.2, the total quantity of each layered material can be calculated using the formula^33^
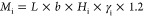
1where *M*_i_ is the total amount of layered material in kg, *L* is the length of the model frame in m, *b* is the width of the model frame in m, *H*_i_ is the layer thickness of the model in m, and γ_i_ is material bulk density in kg/m^2^.

**Table 2:** Similar Model Material Consumption Table

no.	lithology	number ratio	total weight (kg)	sand (kg)	calcium carbonate (kg)	gypsum (kg)	water (kg)	borax (kg)
1	conglomerate	337	923.4	692.5	69.2	161.6	131.9	1.31
2	mudstone	373	33.3	25.0	6.2	2.1	4.8	0.05
3	coal seam	573	12.0	10	1.4	0.6	1.3	0.01
4	silt sandstone	355	138.5	103.9	17.3	17.3	19.8	0.20
5	coal seam	573	18.0	15	2.1	0.9	2	0.02
		373	22.2	16.7	3.9	1.6	3.2	0.03
		373	22.2	16.7	3.9	1.6	3.2	0.03
		373	22.2	16.7	3.9	1.6	3.2	0.03
		373	22.2	16.7	3.9	1.6	3.2	0.03
		373	22.2	16.7	3.9	1.6	3.2	0.03
6	mudstone	373	22.2	16.7	3.9	1.6	3.2	0.03
		373	22.2	16.7	3.9	1.6	3.2	0.03
		373	22.2	16.7	3.9	1.6	3.2	0.03
		373	22.2	16.7	3.9	1.6	3.2	0.03
		373	22.2	16.7	3.9	1.6	3.2	0.03
7	coal seam	573	59.9	49.9	7	3	6.7	0.07
8	mudstone	373	266.4	199.8	46.6	20	38.1	0.38

#### 3.1.2 Model Making

**Figure 2:**
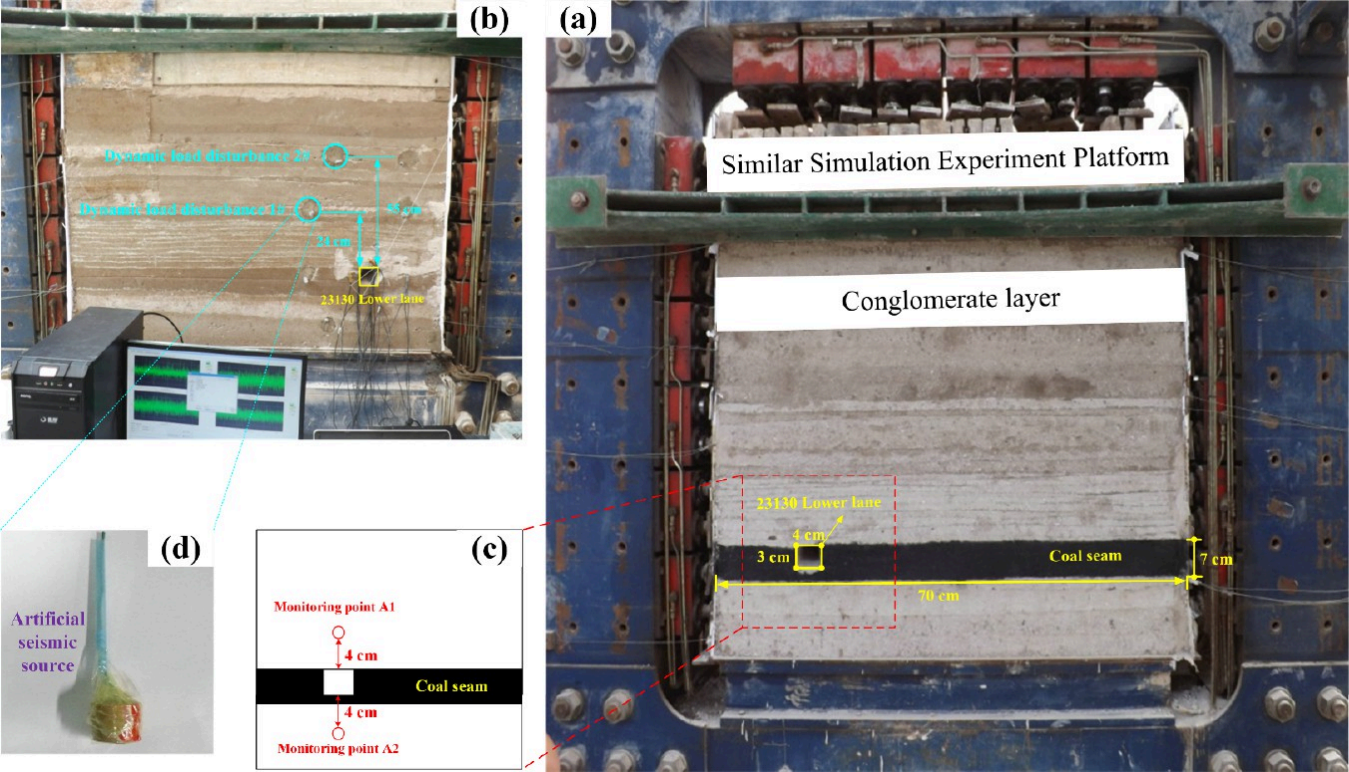
Dynamic load disturbance similar simulation experiment. (a) Similar simulation experiment platform. (b) Arrangement of dynamic load disturbance measuring points. (c) Acceleration monitoring points A1 and A2. (d) Artificial dynamic load disturbance source.

A similar simulation experiment focuses on the inclined direction of the 23130 working face. Due to limitations in the size of the model frame, only one working face can be simulated. The dimensions of the working face are 85 cm in length, 4 cm in width, and 3 cm in height, with the floor coal seam height at 7 cm. The specific layout details of the similar model are illustrated in Figure 2, considering that the original coal-rock mass exists in a three-dimensional stress state. Considering that the strength of the rock mass is lower than that of the rock due to numerous fractures, the strength of the model material is taken as half of the calculated value. Stress similarity parameters determine the upper load on the model. The simulated platform has an overlying rock layer thickness of 730 m, and the actual overlying rock load is *P* = ρ*gh* = 17.88 MPa, where ρ is the simulated platform rock density and *h* is the thickness of the overlying rock layer in the simulated platform. The similar load applied to the model’s top is *P*_m_ = *P*/*C*_σ_ = 0.103 MPa. Furthermore, since the coal-rock mass in the prototype is subjected to triaxial stress. At the same time, a similar simulation test uses a plane stress model, and the strength of the coal-rock mass is significantly reduced. Therefore, the surface force applied at the model’s top should be half the actual value, i.e., 0.052 MPa, applied using lever loading.

#### 3.1.3 Layout of Measuring Points

##### 3.1.3.1 Arrangement of Dynamic Load Disturbance Points and Measuring Points

After the roadway excavation stabilizes, dynamic load disturbances are positioned behind the model. The first dynamic load disturbance point, labeled as 1#, is situated 24 cm from the roof of the coal seam. In contrast, the second dynamic load disturbance point, labeled as 2#, is positioned within the thick conglomerate, 55 cm above the coal seam. Artificial sources are buried inside both dynamic load disturbance points, and high-strength cement is used for sealing. To effectively monitor the influence of dynamic load disturbances on the roof and floor of the 23130 roadway, dynamic acceleration monitoring points, denoted as A1 and A2, are established 4 cm above and below the roadway’s roof and floor, respectively. These monitoring points utilize WS-5921/N60216-C16 dynamic data acquisition instruments, BZ2105-4 charge voltage filter integral amplifiers and data lines, and acceleration sensors. The arrangement of measuring points is illustrated in Figure 2b–d.

##### 3.1.3.2 Experimental Steps

This similar simulation experiment is divided into two main parts:

(1) simulating the working face advancement process according to the working face operating procedures
(2) providing dynamic load disturbance through artificial dynamic load sources and monitoring the internal acceleration patterns of the tunnel roof and floor affected by the dynamic load disturbance

The blasting method is employed to simulate the dynamic load disturbance source for the artificial dynamic load source. Specifically, 5 g of black powder is used as the experimental charge. A fuse is inserted into a tube with a diameter of 0.5 cm, which is then packaged using plastic film and transparent tape. The explosion occurs during the experiment.^34^ According to the literature,^35^ the original waveform of the artificial signal considered mainly resembles the microvibration waveform observed when the roof breaks.

### 3.2 Analysis of Similar Simulation Test Results

#### 3.2.1 Analysis of Dynamic Response Law of Overlying Conglomerate Caving in Working Face Excavation

**Figure 3:**
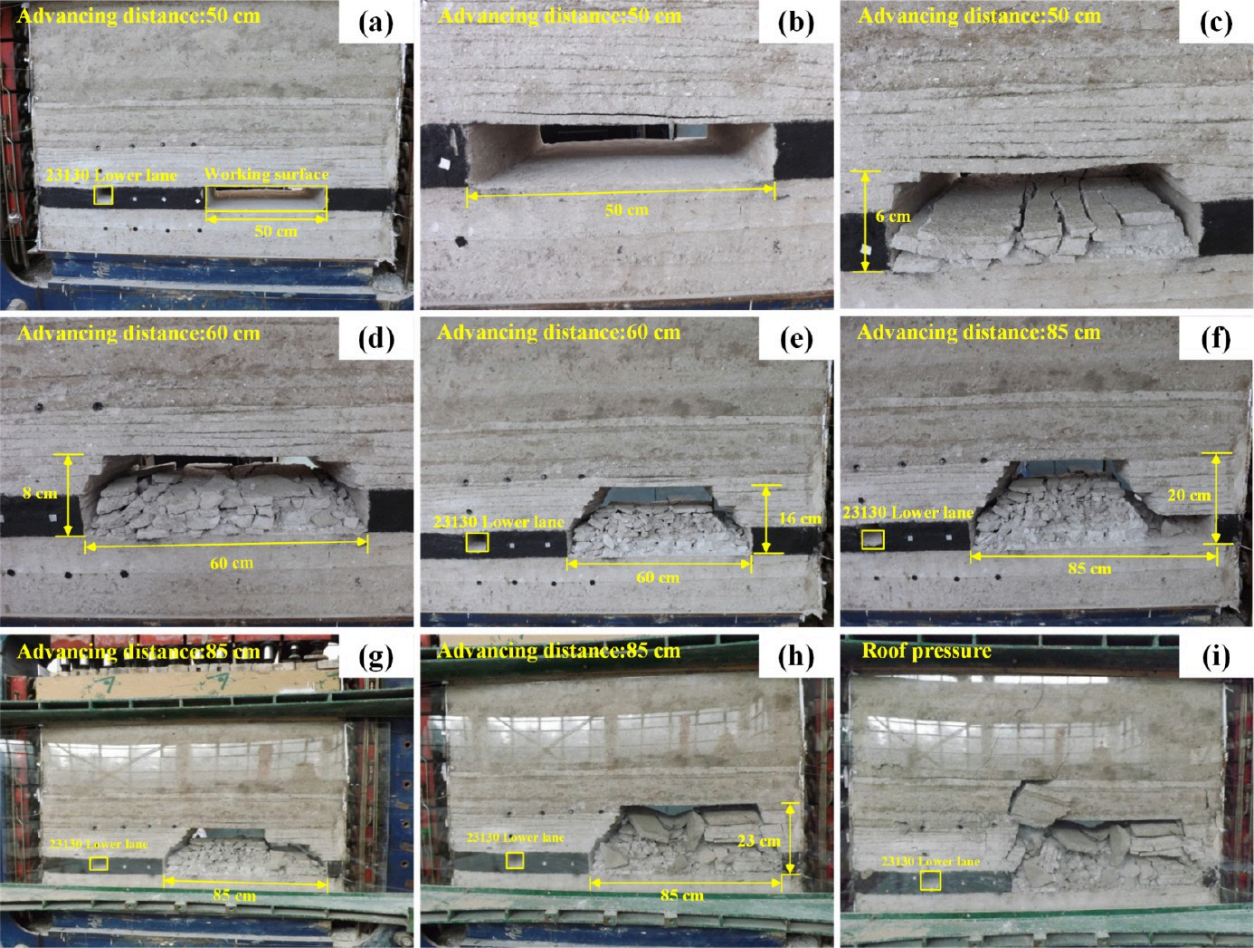
Similar simulation of the overlying rock collapse law. (a–c) The working surface is advanced 50 cm. (d, e) The working surface is advanced 60 cm. (f–h) The working surface is advanced 85 cm. (i) The top plate of the working surface is pressurized.

According to Figure 3, when the working face is mined at 50 cm, the overlying strata on the working face begin to separate. As time goes on, the overlying strata fall by 6 cm under the action of self-weight. When the working face is mined for 60 cm, the overlying strata on the working face further collapses, and the collapse height is 16 cm. When the working face is mined for 85 cm, the final caving height of the overlying strata on the working face is 23 cm. At this time, the working face remains stable and no longer continues to collapse. When the whole model is in a stable state, the pressure is applied to the roof, the overlying strata on the working face further collapses, and a wide range of damage characteristics appear until the model is stable and no change occurs.

#### 3.2.2 Analysis of Acceleration Variation Law of Roadway Roof and Floor under Dynamic Load Disturbance

**Figure 4:**
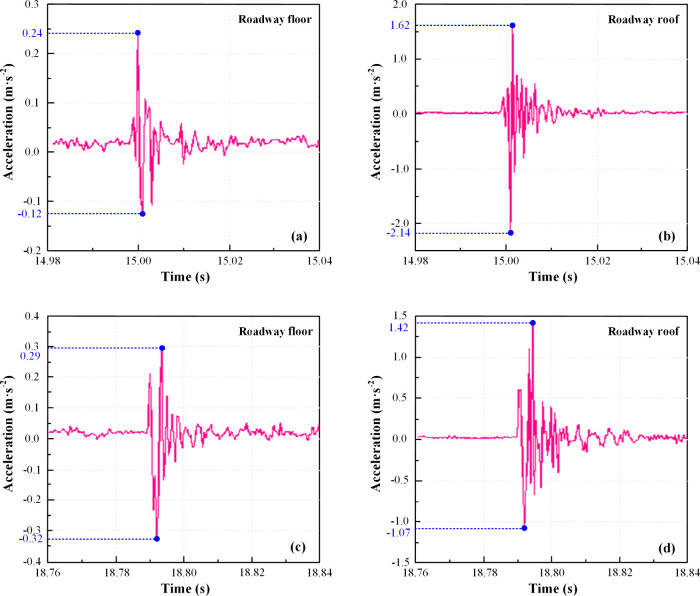
Roadway roof and floor acceleration dynamic law diagram: (a) dynamic load disturbance 1# roadway floor; (b) dynamic load disturbance 1# roadway roof; (c) dynamic load disturbance 2# roadway floor; (d) dynamic load disturbance 2# roadway roof.

Once the working face has been excavated and the internal stress field system of the coal layer has achieved balance and stabilization, the variations in acceleration of the tunnel roof and floor under dynamic load disturbances at different positions are recorded through the acceleration monitoring points A1 and A2, depicted in Figure 4.

Based on Figure 4, it is evident that when subjected to the same dynamic load disturbance, the effect of 1# dynamic load disturbance on the tunnel surpasses that of 2#, with the internal acceleration of the tunnel roof consistently exceeding that of the tunnel floor. When the 1# dynamic load disturbance occurs, the acceleration signal of the tunnel floor fluctuates at 0.02 s, reaching a maximum value of 0.24 m/s^2^ and a minimum value of −0.12 m/s^2^. Meanwhile, the acceleration signal on the tunnel roof simultaneously exhibits a maximum fluctuation of 1.62 m/s^2^ and a minimum of −2.14 m/s^2^. Upon the 2# dynamic load disturbance, the acceleration signal of the tunnel floor fluctuates at 0.03 s, with a maximum value of 0.29 m/s^2^ and a minimum of −0.32 m/s^2^. Concurrently, the acceleration signal on the tunnel roof simultaneously displays a maximum fluctuation of 1.42 m/s^2^ and a minimum of −1.07 m/s^2^. These observations demonstrate that dynamic load disturbances lead to instantaneous increases in acceleration in the coal and rock mass of the roadway roof and floor, followed by a return to equilibrium. Consequently, the coal and rock mass interior experiences sustained extreme static loading. When the dynamic load disturbance point is positioned close to the roadway on the coal seam roof, its impact on the roadway roof is more pronounced than on the floor. Conversely, suppose the dynamic load disturbance point is situated within the conglomerate away from the tunnel. In that case, the resultant vibration wave attenuates during propagation through the conglomerate, leading to a relative decrease in acceleration fluctuation of the tunnel roof. However, the conglomerate’s self-weight amplifies the tunnel floor’s acceleration fluctuation accordingly.

## 4 A Similar Simulation Experiment on the Impact of Dynamic Load Disturbance under Extremely Thick Conglomerate

The FLAC 2D 5.0 finite element numerical simulation software employs the Lagrangian algorithm. It utilizes the finite difference display algorithm to derive time-step solutions for all motion equations within the model, including internal variables. This approach enables the tracking of progressive material destruction during simulations.^36,37^ FLAC incorporates specific constitutive equations tailored to the properties of different materials, allowing for an accurate representation of their mechanical behavior. As a result, FLAC finds extensive application in analyzing stress and displacement fields across various domains, including water conservancy, mining, earthquakes, geology, petroleum, and civil engineering.^38,39^

### 4.1 Numerical Simulation Model

**Figure 5:**
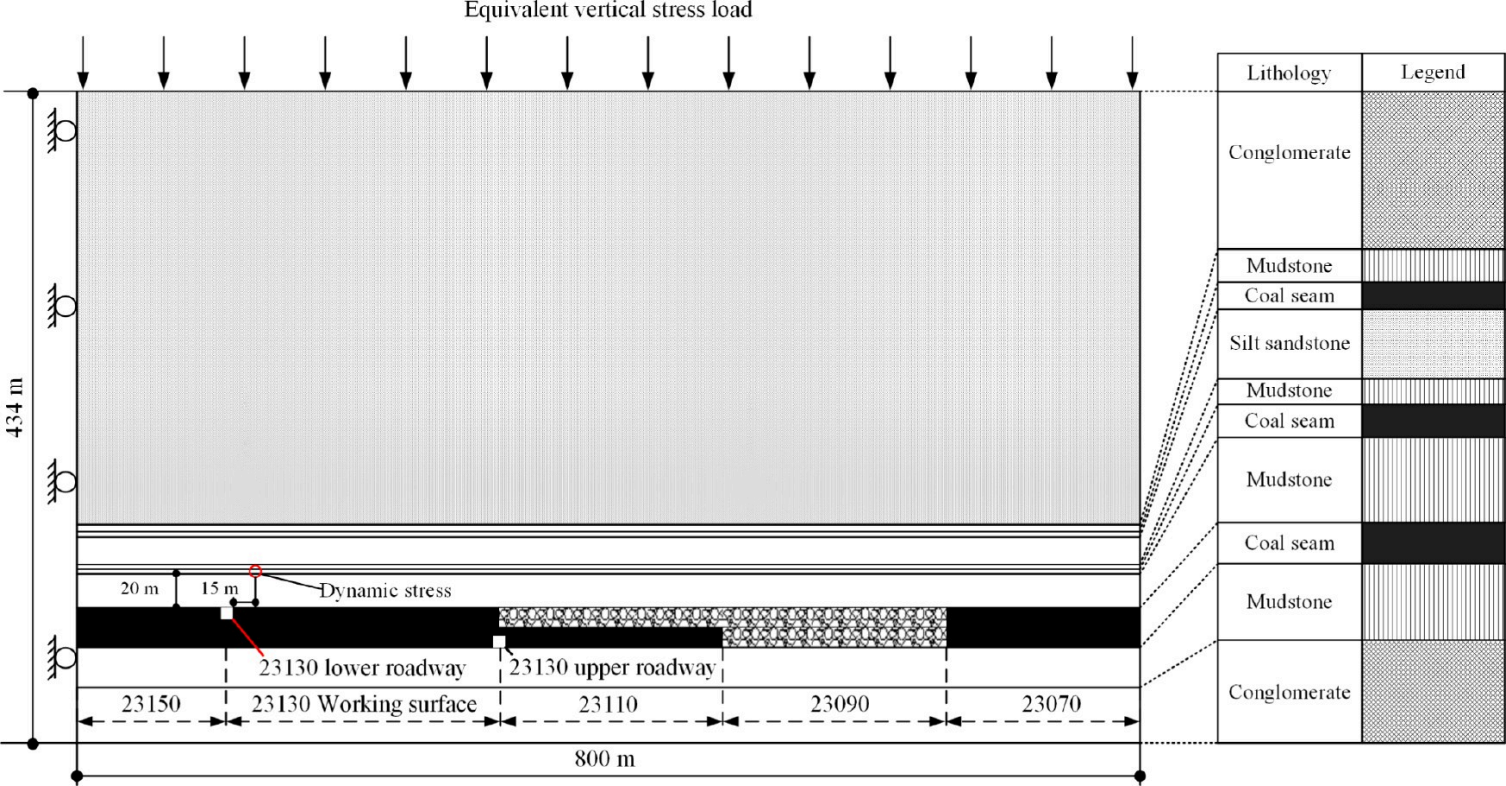
Numerical simulation model overview.

This study will utilize the geological conditions of the 23130 working face as the engineering background, employing the Dynamic module in FLAC 2D 5.0 finite element numerical simulation to construct and simplify the numerical simulation model. The geological conditions will be simplified to investigate the general rules of floor impact mine pressure, considering the coal seam as a gently sloping one. Consequently, the dip angle of the coal layer will not be set in the model. The model dimensions are 800 × 434 m long, comprising 490 × 240 = 117600 units. The dimensions of the coal seam roadway are set as 4 × 3 m, with the excavation area units refined, as shown in Figure 5. Additionally, the unit size within 30 m on both sides of the coal seam and roadway is adjusted to 0.5 × 0.5 m. Based on the conditions between the coal and rock layers, the bedding structure between each coal and rock layer employs the Interface unit to establish a structural plane consistent with the coal and rock layers.

### 4.2 Model Boundary Conditions and Parameters

#### 4.2.1 Boundary Conditions

The numerical model employs plane strain analysis and the Mohr–Coulomb failure criterion. Lateral displacement and velocity at the left and right boundaries are both set to 0, while vertical displacement and velocity at the bottom boundary are also set to 0. In order to meet the stress value of the overlying rock layer with a thickness of 830 m, equivalent vertical stress loads equivalent to 780, 730, 630, and 530 m need to be applied to the top of the model with gravel thicknesses of 50, 100, 200, and 300 m, respectively. The equivalent vertical stress loads are 19.11, 17.88, 15.44, and 12.98 MPa, respectively.^40^ The acceleration due to gravity is assumed to be 9.81 m/s^2^. The rock mass’s self-weight calculates the rock mass’s vertical stress, and the load is σ_*x*_= σ_*y*_ = σ_*z*_. Considering the significant mining depth, the initial stress condition is assumed to be equal in horizontal and vertical directions.

#### 4.2.2 Mechanical Parameters of Coal and Rock Formations

In the numerical simulation process, the mechanical parameters of the coal and rock strata are simplified based on the geological histogram and laboratory test results of boreholes in the 23 mining areas. This simplification aims to streamline the lithology and thickness of the coal and rock strata. The mechanical parameters of the coal and rock mass are detailed in Table 3.

**Table 3:** Mechanical Parameters of Coal and Rock Mass

lithology	thickness (m)	compressive strength (MPa)	elastic modulus (GPa)	Poisson ratio	cohesion (MPa)	internal friction angle (deg)	density (kg/m^3^)
conglomerate	50–300	52.05	42.16	0.2	29	40	2600
mudstone	3	20.00	4	0.35	6	28	2500
coal seam	2	14.43	2	0.4	1.68	25	1350
silt sandstone	12	35.25	26.88	0.15	24.7	35	2600
mudstone	2	20.00	4	0.35	6	28	2500
coal seam	3	14.43	2	0.4	1.68	25	1350
mudstone	20	20.00	4	0.35	6	28	2500
coal seam	9	14.43	2	0.4	1.68	25	1350
mudstone	24	20.00	4	0.35	6	28	2500
conglomerate	50	52.05	42.16	0.2	29	40	2600

### 4.3 Numerical Simulation Plan

In order to investigate the evolution of tunnel floor impact ground pressure under different conglomerate thicknesses, the numerical simulation process is divided into several steps: (1) constructing the initial model, (2) setting the initial stress field, (3) original rock stress balance, (4) mining the 23110 working face, (5) excavation of the tunnel under the 23130 working face, (6) roadway support, implementing support measures, such as anchor rods and anchor cables, to reinforce the tunnel walls and roof after excavation, (7) dynamic load disturbance, and (8) simulation ends.

In the dynamic load disturbance simulation, the shear stress wave is generated 20 m above the roof on the right side of the tunnel. The vibration frequency, action time, peak intensity, and loading type of the dynamic load are specified to replicate real-world conditions accurately. The vibration frequency is set to 20 Hz, the action time to 0.2 s, the peak intensity to 40 MPa, and the loading type to stress time history.

### 4.4 Analysis of Numerical Simulation Results

#### 4.4.1 Stress Cloud Diagram Analysis of Roadway Floor before and after Being Disturbed by Dynamic Load

**Figure 6:**
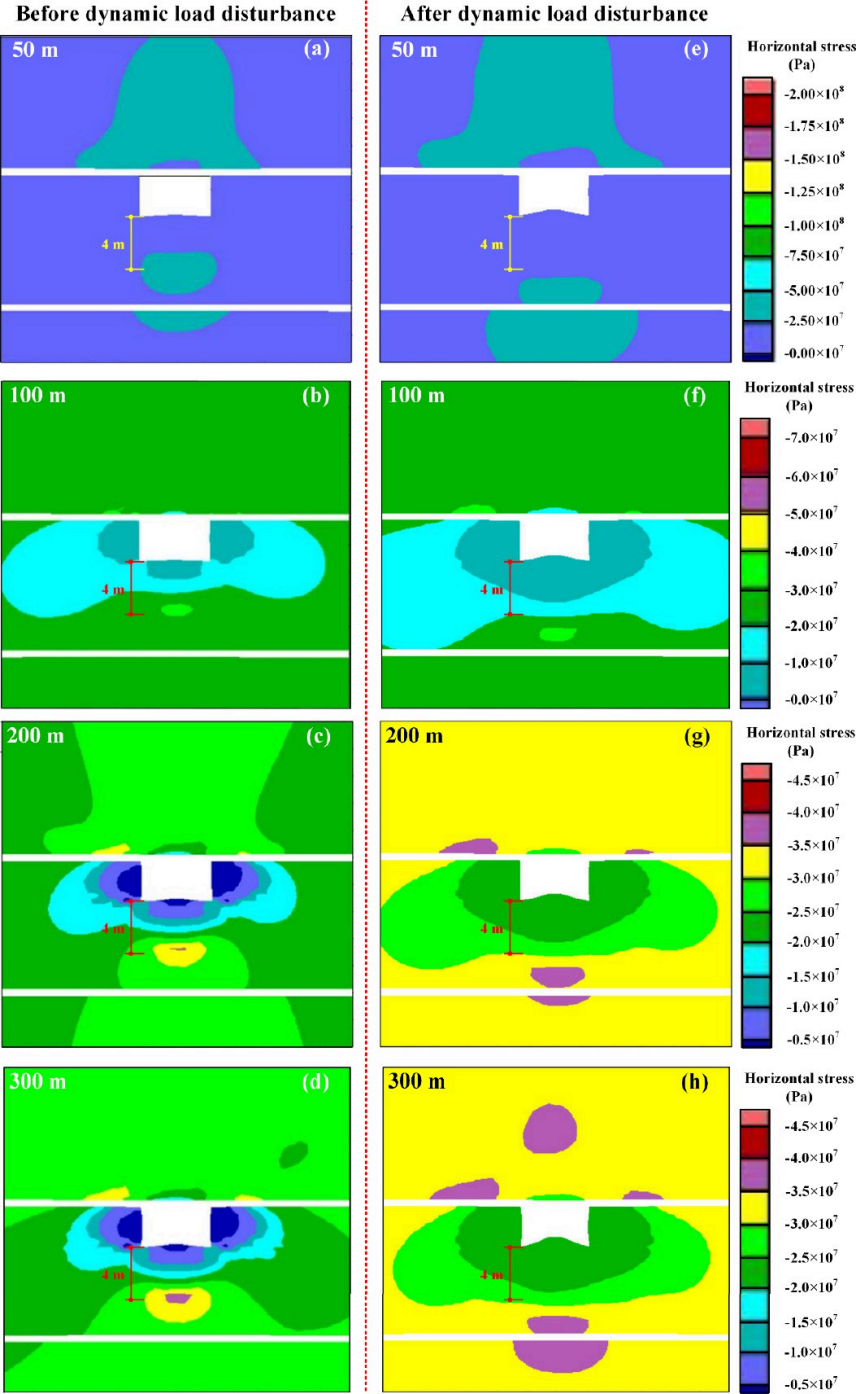
Horizontal stress cloud diagram of roadway before and after dynamic load disturbance: (a–d) before dynamic load disturbance; (e–h) after dynamic load disturbance.

The horizontal stress distribution surrounding the roadway under varying overlying conglomerate thicknesses before and after dynamic load disturbance is illustrated in Figure 6.

According to Figure 6, it is evident that as the thickness of the overlying conglomerate increases, both the intensity and extent of horizontal stress in the surrounding rock of the tunnel gradually escalate under both static and dynamic load conditions. Examining Figure 6a–d, it is apparent that under static loading conditions, the bottom plate experiences notable stress concentration after tunnel excavation, primarily localized around a position 4 m beneath the plate. On the other hand, as depicted in Figure 6e–h, dynamic load disturbance induces significant alterations in the horizontal stress field of the surrounding rock, causing the stress concentration area of the floor to shift from its original position 4 m below to deeper rock strata. When the thickness of the overlying conglomerate reaches 300 m, the analysis reveals a substantial transfer of the horizontal stress concentration area from the roadway floor to extensive regions within the deeper rock formations. Additionally, certain stress concentration areas also emerge on the tunnel roof. These findings indicate that dynamic load disturbance leads to a relocation of stress concentration zones on the tunnel floor. Moreover, as conglomerate thickness increases, energy transfer to the deeper rock formations intensifies, significantly elevating the risk of floor rockbursts.

#### 4.4.2 Stress Changes in Conglomerate Floors of Different Thicknesses under Dynamic Load Disturbance

**Figure 7:**
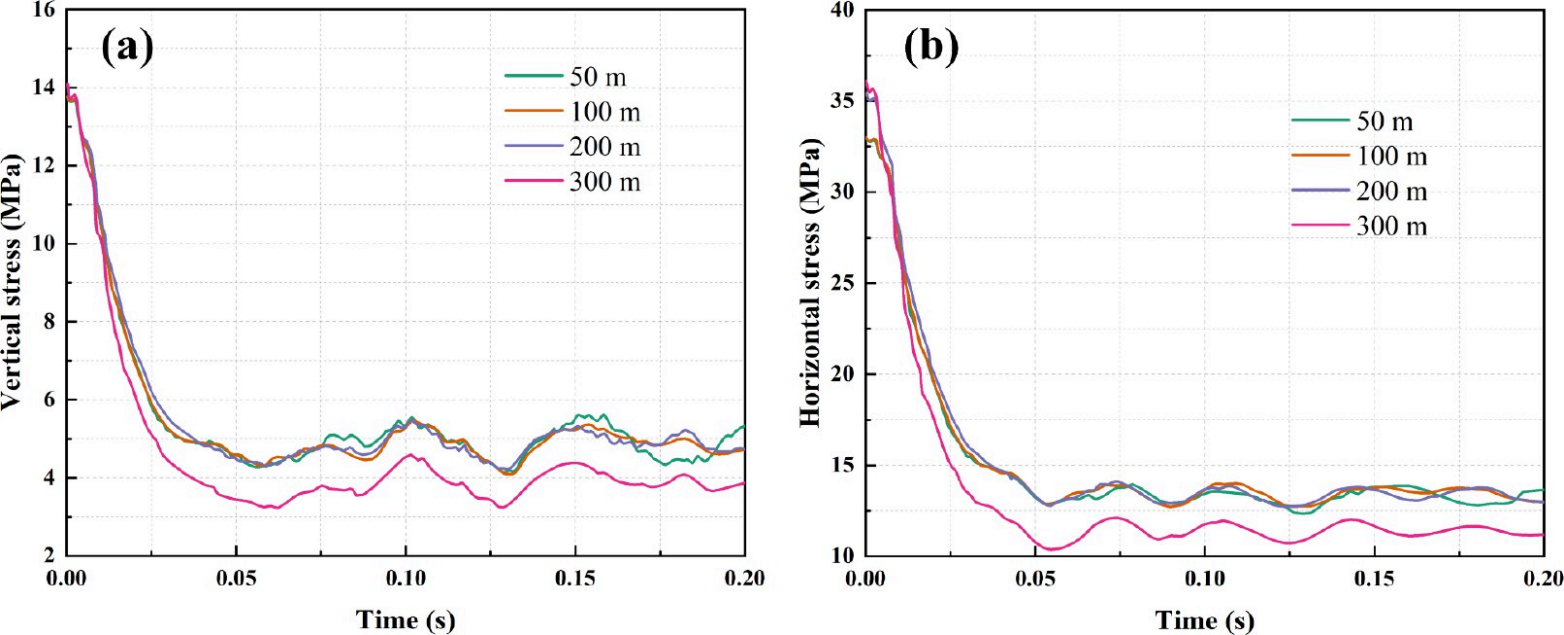
Stress change curve in coal under dynamic load with different conglomerate thicknesses: (a) vertical stress; (b) horizontal stress.

Figure 6 shows that under static loading conditions, stress concentration in the bottom plate of conglomerate tunnels with varying thicknesses primarily occurs approximately 4 m below the bottom plate. In order to obtain the response rules of stress and deformation in the tunnel floor coal seam under the action of dynamic load disturbance, monitoring points were arranged in the floor coal seam at the tunnel excavation position under the 23130 working face, 4 m away from the center of the tunnel. The monitored parameters encompass horizontal stress, vertical displacement, and vertical velocity. The corresponding results are illustrated in Figure 7.

Rockburst, as a manifestation of mine pressure, primarily stems from the instability of localized coal masses and the sudden release of significant deformation energy. Observing Figure 7, it is apparent that following a 0.2 s dynamic load disturbance, the vertical and horizontal stresses at the 4 m position of the tunnel floor gradually diminish, eventually stabilizing. For a conglomerate thickness of 50 m, the vertical stress at the 4 m position of the tunnel floor drops from 13.62 to 5.25 MPa, while the horizontal stress decreases from 32.85 to 13.60 MPa. With a conglomerate thickness of 100 m, the vertical stress at the same position decreases from 13.56 to 4.76 MPa, and the horizontal stress reduces from 32.88 to 13.05 MPa. In the case of a conglomerate thickness of 200 m, the vertical stress at the 4 m position of the tunnel floor decreases from 13.81 to 4.73 MPa, accompanied by a reduction in horizontal stress from 35.40 to 12.99 MPa. Finally, for a conglomerate thickness of 300 m, the vertical stress at the 4 m position of the tunnel floor decreases from 13.86 to 3.85 MPa, with the horizontal stress declining from 35.94 to 11.22 MPa. This trend underscores that with increasing conglomerate thickness, the stress reduction in the tunnel floor becomes more pronounced, amplifying the release of energy.

**Figure 8:**
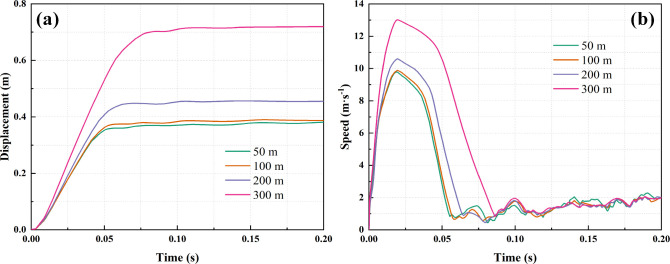
Dynamic response of floor under a dynamic load with different conglomerate thicknesses: (a) displacement; (b) speed.

During the dynamic load disturbance process, the vertical displacement and velocity response of the tunnel floor are depicted in Figure 8. As depicted in Figure 8a, it is evident that with increasing conglomerate thickness, the vertical displacement of the tunnel floor gradually rises. For conglomerate thicknesses of 50, 100, 200, and 300 m, the vertical displacements of the tunnel floor are 0.38, 0.39, 0.46, and 0.72 m, respectively. Figure 8b shows that as the conglomerate thickness gradually increases, the vertical velocity of the tunnel floor initially rises rapidly before declining to an equilibrium position, followed by fluctuations. After dynamic load disturbance, the following occur. With a gravel thickness of 50 m, the vertical velocity peaks at 9.59 m/s at 0.019 s before decreasing to its lowest value at 0.079 s. For a gravel thickness of 100 m, the vertical velocity peaks at 9.69 m/s at 0.019 s, with the lowest value at 0.076 s. With a gravel thickness of 200 m, the vertical velocity peaks at 10.36 m/s at 0.019 s, reaching its lowest value at 0.076 s. With a gravel thickness of 300 m, the vertical velocity peaks at 12.84 m/s at 0.019 s and reaches the lowest value at 0.086 s. Hence, it is apparent that the thicker the conglomerate in the overlying rock layer, the more significant the increase in vertical displacement of the tunnel floor and the longer it takes to complete. Additionally, the higher the peak deformation speed of the tunnel floor, the longer the duration of floor movement. This underscores the significance of conglomerate thickness in influencing tunnel floor rockbursts.

#### 4.4.3 Damage Degree of the Roadway under Dynamic Load Disturbance of Conglomerate of Different Thicknesses

**Figure 9:**
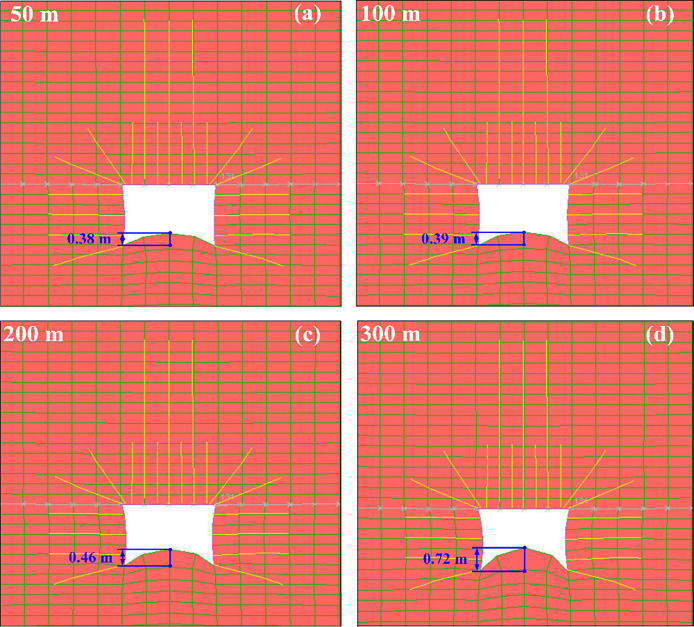
Damage degree of roadway after dynamic load disturbance: (a) conglomerate thickness 50 m; (b) conglomerate thickness 100 m; (c) conglomerate thickness 200 m; (d) conglomerate thickness 300 m.

Under dynamic load disturbance, the degree of damage to the tunnel floor with different overlying conglomerate thicknesses is shown in Figure 9.

As illustrated in Figure 9, it is apparent that as the overlying conglomerate’s thickness increases, the damage to the tunnel floor also escalates. Due to the influence of mining stress on the working face, a substantial horizontal stress concentration area forms at the bottom plate after tunnel excavation. Additionally, the absence of support measures for the tunnel floor leads to impact damage during dynamic load disturbance, resulting in significant changes in floor displacement. When the conglomerate thickness is 50 m, the vertical displacement of the tunnel floor increases by 0.38 m. For a thickness of 100 m, the increase is 0.39 m, for 200 m, it is 0.46 m, and for 300 m, it is 0.72 m, with the most severe damage occurring at this thickness. Effective support measures, such as anchor rods and cables, have been implemented on the roof and sides of the tunnel to mitigate the impact of dynamic load disturbance on the tunnel roof. However, deformation on both sides of the tunnel increases with the thickness of the conglomerate. This underscores that tunnel floor rockbursts are primarily triggered by floor damage and minor damage to the tunnel sides. Moreover, anchor rods and cables effectively control tunnel deformation and damage under dynamic loads.

## 5 Results and Discussion

According to the excavation procedure for the 23130 working face roadway, blasting is employed with a total charge of 12 kg. In the lower roadway of the 23130 working face, 7 mine pressure incidents were recorded. Among these, 6 were attributed to blasting activities, while 1 was induced by the collapse of overlying rock at the working face. The statistics of rock mine pressure events are presented in Table 4.

**Table 4:** Statistical Table of Rockburst Incidents in the Lower Roadway of the 23130 Working Face

serial no.	location	event type	predisposing factors
1	distance from the heading 10–100 m	bottom heaving 1.0–3.5 m	simultaneous blasting
2	distance from the heading 10–100 m	bottom heaving 0.5–1.4 m	simultaneous destressing blasting
3	distance from the heading 10–100 m	bottom heaving 0.3–0.5 m	simultaneous blasting
4	distance from the heading 10–100 m	bottom heaving 0.3–0.6 m	5 min after blasting
5	distance from the heading 10–100 m	bottom heaving 0.2–0.5 m	simultaneous blasting
6	distance from the heading 8–48 m	bottom heaving 0.3–0.5 m	simultaneous blasting
7	310 m from the mining face	bottom heaving 0.5–1.2 m in front of the working face at 340–640 m	roof fracture

**Figure 10:**
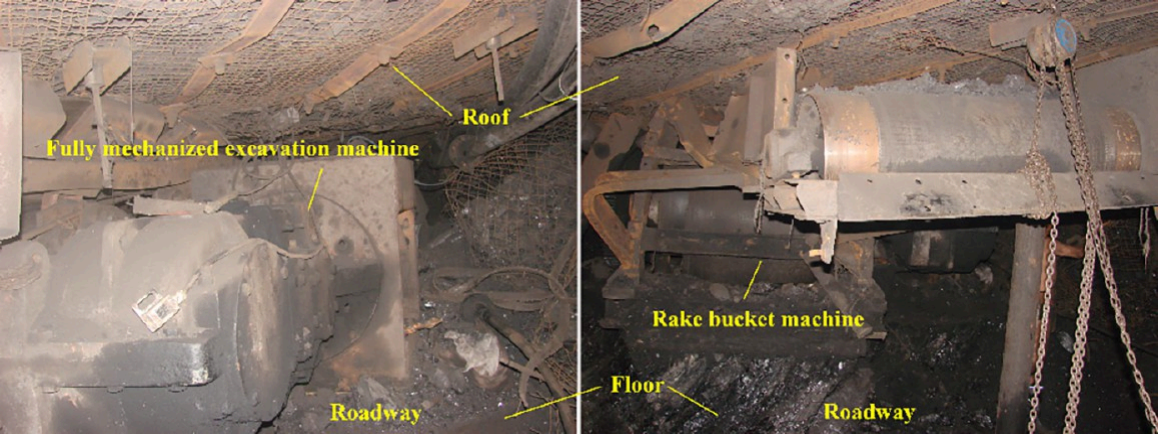
Roadway damage after floor impact.

The rockburst events in the 40 m tunnel between 552 and 592 m were selected for analysis. When a rockburst occurred, a strong shock wave was generated, and the air duct 182 m away from the front ruptured for about 1 m, accompanied by gas gush. 23130 damage condition of the lower tunnel: Starting from 562 m (unloading platform), the tail belt bracket shifted downward by 0.3 m. The mechanized excavator and the random belt rolled over and shifted downward by 1.5 m. The bottom drum phenomenon occurred between 552 and 592 m of the tunnel, and the height of the bottom drum was 0.3–0.5 m. Among them, at the location of the funnel trough of the rake machine, the height of the tunnel under the 23130 working face was only 1.1 m, and the bottom heave of the floor was 1.5 m. The 6 m unerected shed section between shed no. 695 and the rake machine had an upper shear of 2.0 m and a lower shear of 0.5 m, as shown in Figure 10. According to Figure 10, it can be seen that the rise of the tunnel floor causes severe damage, while the impact of the roof is relatively small. This shows that this study’s theory of tunnel floor impact disaster induced by dynamic load disturbances such as blasting and roof fracture is consistent with the actual situation on site.

## 6 Conclusions

This study utilizes the 23130 working faces of Yuejin Coal Mine in Yima Coalfield as its engineering context, employing similar simulation experiments and finite element numerical simulation to investigate the failure patterns of tunnel floors induced by dynamic load disturbances under extremely thick conglomerate layers. The key conclusions drawn are as follows.

(1) Analysis of similar simulation experiments reveals that dynamic load disturbances lead to instantaneous acceleration increments in tunnel roof and floor coal and rock mass, subsequently declining to a stable state. This perpetuates the coal and rock mass within the roadway to endure prolonged periods of high static loading. The proximity of dynamic load disturbance points to the tunnel accentuates their impact on the roof relative to the floor. Conversely, when these points are distant from the tunnel, vibration wave attenuation occurs during conglomerate propagation, reducing roof acceleration fluctuations. However, floor acceleration fluctuations amplify accordingly, congruent with the conglomerate’s self-weight.
(2) Examination of finite element FLAC 2D numerical simulation results underscores the pivotal role of conglomerate thickness in governing tunnel floor rockbursts. As conglomerate thickness escalates, the stress concentration zone within the tunnel floor progressively shifts toward deeper rock layers. This escalation in stress reduction trends correlates with heightened energy release and augmented vertical displacement of the tunnel floor. Moreover, effective control over tunnel deformation and damage under dynamic load exposure is feasible by applying support measures such as anchor rods and cables.
(3) Integrating field observations, the theoretical framework positing tunnel floor impacts induced by dynamic load disturbances under extremely thick conglomerates aligns with empirical realities. This underscores the critical role of dynamic load disturbances like blasting and roof collapse in instigating tunnel floor damage, offering foundational insights for analogous coalfield operations.
